# HEMA but not TEGDMA induces autophagy in human gingival fibroblasts

**DOI:** 10.3389/fphys.2015.00275

**Published:** 2015-10-02

**Authors:** Gabriella Teti, Giovanna Orsini, Viviana Salvatore, Stefano Focaroli, Maria C. Mazzotti, Alessandra Ruggeri, Monica Mattioli-Belmonte, Mirella Falconi

**Affiliations:** ^1^Department of Biomedical and Neuromotor Sciences, University of BolognaItaly; ^2^Department of Clinical Sciences and Stomatology, Polytechnic University of MarcheAncona, Italy; ^3^Department of Medical and Surgical Sciences, University of BolognaItaly; ^4^Department of Clinical and Molecular Sciences, Polytechnic University of MarcheAncona, Italy

**Keywords:** dental resin monomers, autophagy, apoptosis, human gingival fibroblasts, adaptive cell response

## Abstract

Polymerized resin-based materials are successfully used in restorative dentistry. Despite their growing popularity, one drawback is the release of monomers from the polymerized matrix due to an incomplete polymerization or degradation processes. Released monomers are responsible for several adverse effects in the surrounding biological tissues, inducing high levels of oxidative stress. Reactive oxygen species are important signaling molecules that regulate many signal-trasduction pathways and play critical roles in cell survival, death, and immune defenses. Reactive oxygen species were recently shown to activate autophagy as a mechanism of cell survival and cell death. Although the toxicity induced by dental resin monomers is widely studied, the cellular mechanisms underlying these phenomena are still unknown. The aim of the study was to investigate the behavior of human gingival cells exposed to 2-hydroxy-ethyl methacrylate (HEMA) and triethylene glycol dimethacrylate (TEGDMA) to better elucidate the mechanisms of cell survival and cell death induced by resin monomers. Primary culture of human gingival cells were exposed to 3 mmol/L of HEMA or 3 mmol/L of TEGDMA for 24, 48, and 72 h. Morphological investigations were performed by transmission electron microscopy to analyze the ultrastructure of cells exposed to the monomers. The expression of protein markers for apoptosis (caspase – 3 and PARP) and autophagy (beclin – 1 and LC3B I/II) were analyzed by western blot to investigate the influence of dental resin monomers on mechanisms underlying cell death. Results showed that HEMA treatment clearly induced autophagy followed by apoptosis while the lack of any sign of autophagy activation is observed in HGFs exposed to TEGDMA. These data indicate that cells respond to monomer-induced stress by the differential induction of adaptive mechanisms to maintain cellular homeostasis.

## Introduction

The development of new generations of resin-based dental restorative materials has allowed for the application of more conservative, esthetic, and long lasting restorative techniques. These adhesive techniques are extensively used in a wide variety of applications in dentistry, including restorative procedures, prosthodontics, orthodontics and preventive dentistry, making resin-based composites one of the most important groups of materials in dental practice (Bakopoulou et al., 2009). Most of these products consist of a mixture of various methacrylate monomers, such as BisGMA (2,2-bis[4-(2-hydroxy-3- methacryloxypropoxy)phenyl]propane) and UDMA (urethane dimethacrylate) in combination with comonomers of lower viscosity, such as TEGDMA (triethyleneglycol dimethacrylate), EGDMA (ethyleneglycol dimethacrylate), or DEGDMA (diethyleneglycol dimethacrylate) (Peutzfeldt, 1997; Ilie and Hickel, 2011).

These methacrylate monomers are responsible for major clinical disadvantages, such as polymerization shrinkage of the composites leading to microleakage phenomena in the tooth-material interface (Braga et al., 2005), as well as adverse effects caused by substances released from the resinous matrix due to incomplete polymerization or resin degradation (Schweikl et al., 2006, 2007; Bakopoulou et al., 2009). Incomplete polymerization and the leaching of monomers not only decrease the mechanical properties of a restoration, but can also negatively impact the biocompatibility of the materials (Bakopoulou et al., 2009). Based on *in vitro* cell culture experiments of multiple target cells, resin monomers, such as 2-hydroxyethyl methacrylate (HEMA) and TEGDMA, were shown to specifically interfere with various vital cellular functions (Schweikl et al., 2006; Krifka et al., 2013). Dental resin monomers cause persistent inflammatory responses (Schmalz et al., 2011), down-regulate several extracellular matrix proteins (Falconi et al., 2007, 2010; Zago et al., 2008; Teti et al., 2009), disturb reparative dentinogenesis and reduce the expression of genes involved in biomineralization (About et al., 2002; Galler et al., 2011).

HEMA and TEGDMA are a likely cause of cellular stress via the formation of reactive oxygen species (ROS) (Stanislawski et al., 2003; Chang et al., 2005). Resin monomers deplete the amount of intracellular antioxidant glutathione (GSH), while in parallel increasing the formation of ROS (Chang et al., 2005) thereby inducing cell death via apoptosis, delayed cell proliferation and mineralization processes (Schweikl et al., 2007). The cellular mechanisms underlying these phenomena remain poorly understood.

Autophagy is a catabolic process aimed at recycling cellular components and damaged organelles in response to diverse conditions of stress, such as nutrient deprivation, viral infection and genotoxic stress. A growing amount of evidence in recent years argues for oxidative stress acting as the converging point of these stimuli, with ROS and reactive nitrogen species (RNS) being among the main intracellular signal transducers sustaining autophagy (Filomeni et al., 2015).

The autophagy pathway is based on distinct steps, including induction, vesicle nucleation, selective cargo recognition, autophagosome formation, autophagosome-lysosome fusion, cargo degradation, and nutrient recycling (Huang et al., 2011). More than 30 key components of the autophagy machinery encoded by autophagy-related genes (ATGs) function at different steps of this process. Beclin – 1 protein represents a primary cellular activator of autophagy involved in the autophagosome initiation and assembly (Huang et al., 2011), while microtubule associated protein light chain 3 (LC3) is mainly involved in the elongation of the autophagosome. Endogenous LC3 is detected as two bands following SDS-PAGE and immunoblotting: one represents LC3-I, which is cytosolic, and the other LC3-II, which is conjugated with autophagosomes (Parzych and Klionsky, 2014).

The aim of this study was to verify an involvement of autophagy in human gingival fibroblasts exposed to HEMA and TEGDMA. Cell viability data, transmission electron microscopy and western blotting were carried out to this aim. The main goal is to demonstrate a further adaptive cell response to oxidative stress caused by monomers, for a better understanding of the mechanisms involved in toxicity induced by resin dental materials.

## Materials and methods

### Human gingival fibroblasts (HGFs)

HGFs were obtained from healthy patients subjected to gingivectomy of the molar region. Informed consent was obtained from each patient. Immediately after removal, the tissues were washed in phosphate buffer, cut in small pieces and placed in Dulbecco's Modified Eagles' Medium (DMEM) (Life Technologies, Milan, Italy), supplemented with 10% fetal calf serum (FCS), penicillin (50 UI mL^−1^), and streptomycin (0.05 mg mL^−1^), at 37°C in a 5% humidified CO_2_ atmosphere. After the first passage, the HGFs were routinely cultured in DMEM supplemented with 10% FCS and were not used beyond the fifth passage.

### Cell viability assay

HGFs were seeded into 96-well plates (2 × 10^4^/well) for 24 h. Then, the medium was changed with a new one containing different concentrations of HEMA or TEGDMA and incubated for 24 h. Cell viability was established using the resazurin-based reagent cell viability reagent (PrestoBlue; Life Technologies, Grand Island, NY) diluted at 1:20 with the culture medium, allowing incubation of the reaction for 2 h at 37°C. The optical density in each well was immediately measured using a spectrophotometer Microplate Reader (Model 680, Biorad Lab Inc., CA, USA) at a wavelength of 570 nm. Each experiment was performed three times, and four replicate cell cultures were analyzed in each experiment. The optical density obtained from treated cell cultures was expressed as the percentage of untreated cells.

### HEMA and TEGDMA treatment

HEMA and TEGDMA were previously dissolved in ethanol as solvent at a concentration of 1 mol/L (stock solution). Subsequently, HGFs were treated with 3 mmol/L HEMA or 3 mmol/L of TEGDMA for 24, 48, and 72 h.

For each concentration of HEMA and TEGDMA treatment, the final concentration of ethanol used for cell incubation was no more than 0.5%, which corresponds to a nontoxic concentration (Falconi et al., 2007). Controls consisted in untreated HGFs and HGFs exposed to the same concentration of solvent for the previously described periods of time.

### Transmission electron microscopy (TEM)

HGFs were initially collected after each experimental point, and fixed with 2.5% glutaraldehyde in 0.1 M cacodylate buffer for 2 h and post fixed with a solution of 1% osmium tetroxide in 0.1 M cacodylate buffer. The cells were then embedded in epoxy resins after a graded-ethanol serial dehydration step. The embedded cells were sectioned into ultrathin slices, stained by uranyl acetate solution and lead citrate, and then observed with transmission electron microscope CM10 Philips (FEI Company, Eindhoven, The Netherlands) at an accelerating voltage of 80 kV. Images were recorded by Megaview III digital camera (FEI Company, Eindhoven, The Netherlands).

### Protein extraction and western blot analysis

After each experimental point, HGFs were washed with PBS, collected and lysed in RIPA buffer (Pierce, Life technologies, Milan, Italy) containing protease inhibitor cocktail. Proteins (50μg/sample) were separated by 4-12% SDS/PAGE and transferred to a nitrocellulose membrane (GE Healthcare Europe GmbH, Milan, Italy). After being blocked with 5% skim milk powder diluted in TBS containing 5% Tween-20 for 30 min, the membrane was incubated with a primary antibody, anti-caspase -3 (Cell Signaling Technology, Danvers, MA, USA), anti- beclin -1 (Cell Signaling Technology, Danvers, MA, USA), anti-LC3B I-II (Cell Signaling Technology, Danvers, MA, USA), anti-PARP (Santa Cruz Biotecnology, Inc, Santa Cruz, California), and anti β-tubulin (Sigma Aldrich, St. Louis, Missouri, USA) at 4°C overnight. Immunoreactive proteins were detected using an enhanced chemiluminescence light (ECL) detecting kit (GE Healthcare Europe GmbH, Milan, Italy). β-tubulin acted as a loading control. Images were obtained by Image Station 2000R (Kodak, New York, New York). The experiments were replicated at least three times, and representative results are shown.

### Statistical analysis

Statistical differences were assessed by One-Way ANOVA (*P* < 0.05) and Dunnett multiple comparison test (*P* < 0.05). The statistical analysis was performed with GraphPad Prism 5.0 software.

## Results

### Cell viability assay

**Figure 1:**
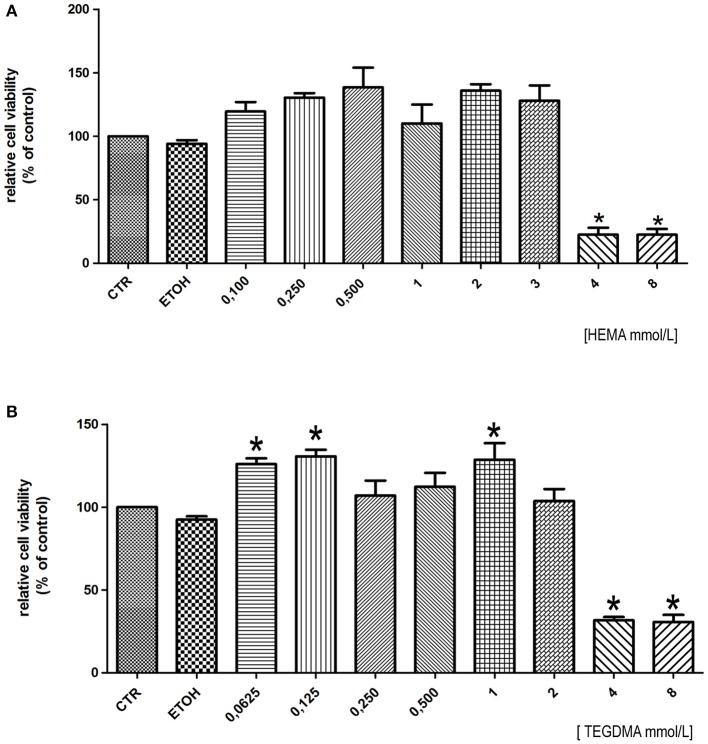
**Cell viability assay of HGFs exposed to different concentration of HEMA (A) and TEGDMA (B) for 24 h**. Data were expressed as relative percentage ± SD compared to control HGFs (CTR). ^*^Represents a significant difference compared to CTR HGFs, *P* < 0.05.

To establish the concentration of HEMA and TEGDMA to test in this study we firstly performed baseline dose–response curves to HEMA and TEGDMA for the human gingival cells. In HGFs exposed to HEMA for 24 h, a strong reduction in cell viability was observed at 4 and 8 mmol/L (Figure 1A) (IC50: 3.79 mmoL/L). A similar pattern was observed in HGFs exposed to different doses of TEGDMA where 4 and 8 mmol/L (IC50: 3.46 mmol/L) represented the concentrations to which the HGFs were extremely susceptible (Figure 1B). We decided to perform the following experiments testing the concentration of 3 mmoL/L for both resins, values below the respective IC50.

### TEM analysis for HGFs exposed to HEMA

**Figure 2:**
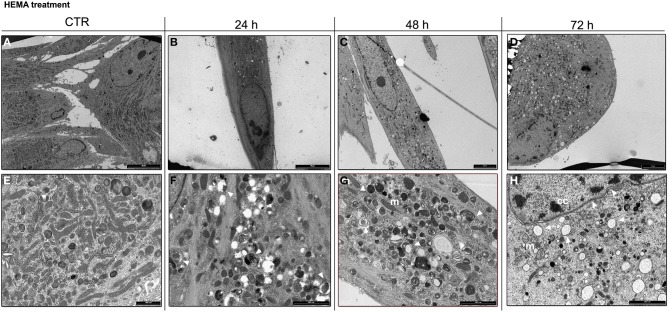
**(A)** Control HGFs showing a well preserved morphology (bar: 10 um); **(E)** Detail of cytoplasm in control cells showing several mitochondria with a regular shape (bar: 1 um); **(B)** HGFs exposed to 3 mmol/L of HEMA for 24 h. Cells showed a preserved fibroblast morphology (bar: 10 um); **(F)** few autophagic vesicles (arrowhead) detected in the cytoplasm of HGFs treated for 24 h (bar: 2 um); **(C)** HGFs exposed to HEMA for 48 h. Nucleus is still well preserved (bar: 5 μm); **(G)** higher magnification of cytoplasm in HGFs exposed to HEMA for 48 h. Several autophagic vesicles (arrowhead) were detected. Mitochondria (m) still showed a regular shape (bar: 2.5 um); **(D)** HGFs exposed for 72 h. Cells appear round shaped and the nucleus showed masses of condensed chromatin (cc) (bar: 5 μm); **(H)** higher magnification of cytoplasm in HGFs exposed for 72 h. Dilated nucleus envelope (arrowhead) and enlarged RER (arrow) were detectable. Mitochondria (m) showed a degraded morphology (^*^) (bar: 2.5 um).

Ultrastructural analysis showed a preserved morphology in untreated HGFs, where nucleus and cytoplasmic organelles were easily detectable (Figure 2A). Control HGFs were characterized by several mitochondria scattered in the cytoplasm (Figure 2E). Cells exposed to 3 mmol/L of HEMA for 24 h showed a fibroblast like morphology, with the nucleus well preserved (Figure 2B). Some autophagic vesicles and empty vacuoles characterized the cytoplasm (Figure 2F). After 48 h of exposition, cells still showed a well preserved morphology, while in the cytoplasm a higher number of autophagic vesicles was detected (Figures 2C,G). Mitochondria were well preserved. At 72 h of HEMA exposition, cells showed a round or polygonal morphology (Figure 2D) with cytoplasm characterized by several autophagic vesicles. At higher magnification, condensed masses of chromatin were observed in the nucleus and the nuclear envelope was enlarged (Figure 2H). In the cytoplasm, several autophagic vesicles, swollen mitochondria and dilated rough endoplasmic reticulum (RER) were detectable (Figure 2H).

### Apoptosis and autophagy in HGFs exposed to 3 mmol/L of HEMA

**Figure 3:**
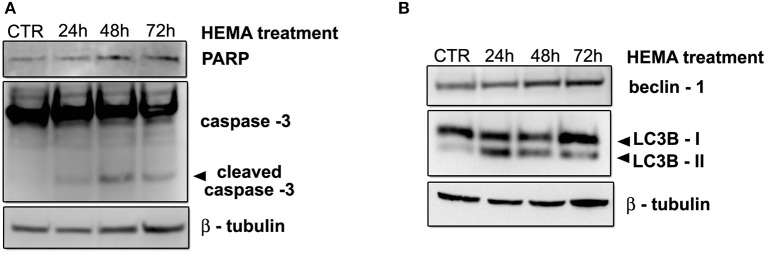
**(A)** Western blot analysis of caspase -3 and PARP in HGFs treated with 3 mmol/L HEMA for 24, 48, 72 h. **(B)** Western blot analysis for Beclin-1 and LC3B I/II expression in HGFs exposed to 3 mmol/L HEMA for the indicated times. β—tubulin represents equal lane loading.

To better evaluate the presence of apoptosis and autophagy in HGFs exposed to HEMA, the expression of protein markers for apoptosis (caspase – 3 and PARP) and autophagy (beclin – 1 and LC3B I/II) were analyzed by western blot. Results showed the activation of caspase – 3 (cleaved caspase -3) after 72 h of treatment, and no sign of PARP activation in all the experimental points (Figure 3A). On the contrary, Western blot analysis demonstrated an increase in the expression of both beclin-1 and the fast migrating form of LC3B (LC3B II, 14 kDa), while LCB I (16 kDa) decreased (Figure 3B), suggesting the activation of the autophagic pathway.

### TEM analysis for HGFs exposed to TEGDMA

**Figure 4:**
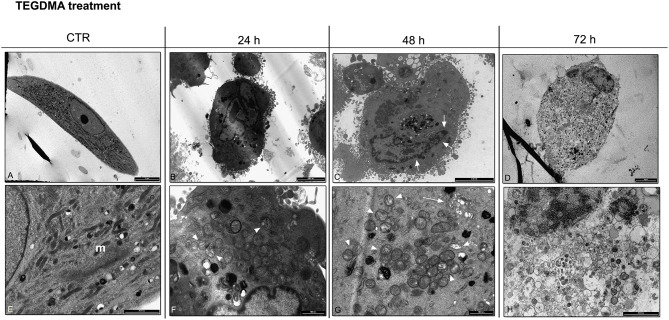
**(A)** TEM image showing untreated HGFs with a well preserved morphology (bar: 10 um); **(E)** Detail of cytoplasm in control cells showing several mitochondria (m) with a regular shape (bar: 2 um); **(B)** HGFs exposed to 3 mmol/L of TEGDMA for 24 h. Cells showed a round shape morphology (bar: 5 um); **(F)** in the cytoplasm of HGFs treated for 24 h damage mitochondria (arrowhead) were detected (bar: 1 um). No autophagy vesicles were observed; **(C)** HGFs exposed to TEGDMA for 48 h. Chromatin condensation (arrow) was detected in the nucleus (bar: 5 um); **(G)** higher magnification of cytoplasm in HGFs exposed to TEGDMA for 48 h. Several damaged mitochondria (arrowhead) and an enlargement in Golgi apparatus (arrow) were observed (bar: 1 um); **(D)** Necrotic HGFs exposed for 72 h to 3 mmol/L of TEGDMA (bar: 10 um); **(H)** higher magnification of cytoplasm in HGFs exposed for 72 h. No cytoplasmic organelles were distinguishable (bar: 2 um).

TEM images showed untreated HGFs with a fibroblastic shape and a well preserved morphology. Regular cytoplasmic organelles were detected (Figures 4A,E). After 24 h of TEGDMA treatment, cells were slightly damaged (Figure 4B). The nucleus was still well preserved, while the cytoplasm was characterized by the presence of some damaged mitochondria and the lack of autophagic vesicles (Figure 4F). After 48 h of TEGMDA treatment, masses of condensed chromatin were observed (Figure 4C). Several damaged mitochondria and an enlarged Golgi apparatus were detected in the cytoplasm (Figure 4G). After 72 h of TEGDMA exposition, cells showed a clear morphological pattern of necrosis (Figure 4D). Cytoplasmic organelles were not distinguishable (Figure 4H).

### Apoptosis and autophagy in HGFs exposed to 3 mmol/L of TEGDMA

**Figure 5:**
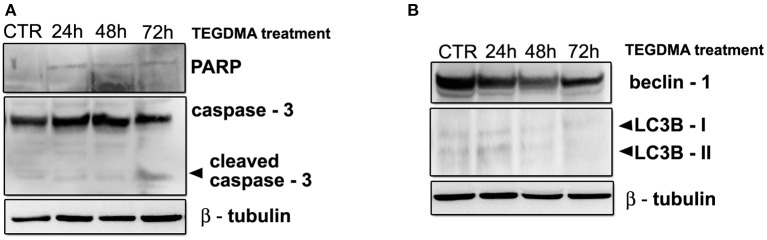
**(A)** Western blot analysis of caspase -3 and PARP in HGFs treated with 3 mmol/L TEGDMA for 24, 48, 72 h. **(B)** Western blot analysis for Beclin-1 and LC3B I/II expression in HGFs exposed to 3 mmol/L TEGDMA for the indicated times. β—tubulin represents equal lane loading.

As previously described for HEMA, to better evaluate the presence of apoptosis and autophagy in HGFs exposed to TEGDMA, the expression of caspase – 3, PARP, beclin – 1 and LC3B I/II were analyzed by western blot. Results showed a light band corresponding to the cleaved fragment of caspase -3 in samples treated for 72 h, and no sign of PARP activation in all the experimental points (Figure 5A). Regarding autophagy, bands corresponding to the up-regulation of beclin-1 and of the fast migrating form of LC3B and LCB I were not observed (Figure 5B).

## Discussion

Dental resin composite are biomaterials commonly used to aesthetically restore the structure and function of teeth impaired by caries, erosion, or fracture (Krifka et al., 2013). Despite their growing popularity, there are concerns that resin-based materials may be toxic, based on the fact that they may release unpolymerized monomers, additives, and filler components in the oral environment (Van Landuyt et al., 2011; Reichl et al., 2012) responsible of adverse effects on cells and tissues nearby (Schweikl et al., 2006, 2007).

The cellular and molecular mechanisms underlying toxicity are still under discussion. Krifka et al. (2013) suggested the presence of multiple adaptive mechanisms in cell responses toward stress caused by dental resin monomers, but the role of autophagy induced by dental resin monomers as a mechanism of cell survival and/or cell death is not well understood.

The aim of the present study was to investigate the involvement of autophagy as a further adaptive mechanism against cell death in HGFs exposed to HEMA and TEGDMA.

The quantity of released dental monomers a patient may be exposed has yet to be determined.

Briefly, the rationale by which the authors have chosen the concentration of HEMA and TEGDMA is based on previous scientific reports, demonstrating that the direct contact of composite materials to dental pulp may induce local adverse effect to the dentin—pulp complex (Modena et al., 2009), even if the release of HEMA and TEGDMA has been estimated to be in micromolar and nanomolar concentrations, amounts far below levels required to cause systemic adverse effects (Van Landuyt et al., 2011; Michelsen et al., 2012). However, due to a great variety in analytical methodology employed in different studies, there is no agreement about the quantities detected in eluates of multiple composite filling materials (Nocca et al., 2011; Van Landuyt et al., 2011; Krifka et al., 2013; Botsali et al., 2014) and further studies are needed to quantify the exact amount of resin monomers release.

According to our previous works (Falconi et al., 2007; Zago et al., 2008; Teti et al., 2009) and cell viability results obtained in this study, we decided to perform all experiments at the concentration of 3 mmol/L for both monomers. These concentrations are just below the IC50 calculated for HEMA and TEGDMA.

Current findings strongly suggest that monomers enhance the formation of ROS, which is most likely the cause of cell death by apoptosis activated by dental composites and resin monomers (Stanislawski et al., 2003). Our data on ultrastructural analysis of HGFs exposed to HEMA up to 72 h demonstrated morphological features more connected with autophagy rather than apoptosis. Indeed, the presence of several intracellular double/multimembranus vesicles, called autophagosomes, which sequester cytoplasms, proteins and organelles are morphological hallmarks of autophagy (Edinger and Thompson, 2004). No ultrastructural features connected with apoptosis such as characteristic chromatin condensation and margination, as well as apoptotic bodies, (Saraste and Pulkki, 2000; Burattini et al., 2010) were detected. By the time of 72 h, damaged mitochondria, enlargement of RER and nuclear envelope suggested the presence of oxidative stress and endothelial reticulum stress (Burattini et al., 2010). The presence of autophagy in HGFs exposed to HEMA was confirmed by western blot data, in which the upregulation of beclin -1, LC3B-II and the down-regulation of LC3B-I were observed in all experimental conditions, while the activation of caspase -3 was detected only after 72 h of treatment. All these results suggest an early attempt of cells to survive by autophagy induction, followed by apoptosis after long term exposition (72 h).

Autophagy is foremost a survival mechanism that is activated in cells subjected to nutrient or obligate growth factor deprivation. When cellular stress continues, cell death may continue by autophagy alone, or else it often becomes associated with features of apoptotic or necrotic cell death (Edinger and Thompson, 2004; Krysko et al., 2008).

HGFs exposed to TEGDMA did not show a similar pattern. Electron microscopy images underlined morphological features connected with necrosis. A few morphological details connected with apoptosis, such as chromatin condensation, were observed just after 48 h of treatment (Burattini et al., 2010). Furthermore, a light band corresponding to the cleaved form of caspase – 3 was detected after 72 h of exposition. Regarding autophagy, there were no upregulation of beclin -1 protein and LC3B-II, suggesting the lack of this phenomena in cells treated with TEGDMA.

In recent years a growing amount of evidence suggests that reactive ROS and reactive nitrogen species (RNS) are among the main intracellular signal transducers sustaining autophagy (Filomeni et al., 2015). Oxidative stress induced by monomers as a result of elevated ROS generation apparently acts as a signal for the activation of pathways which control cell survival and death through the redox-sensitive activation of genes and antioxidant proteins (Krifka et al., 2013). In our experimental conditions, we believe elevated amounts of ROS induced by HEMA are responsible for the activation of autophagy as an adaptive mechanism for cells to survive. On the contrary, ROS do not activate autophagy in HGFs exposed to TEGDMA but exert cytotoxicity via apoptosis and more deeply via necrosis (Krysko et al., 2008).

In conclusion, our data indicate that cells actively respond to monomer-induced oxidative stress by the differential induction of cell death mechanism (autophagy, apoptosis and necrosis) to maintain cellular homeostasis and vital cell functions. Further experiments to investigate the exact molecular mechanisms involved in the induction of autophagy by HEMA treatment and the lack of such response in TEGDMA treated cells are in progress.

## Authorship

Each author substantially contributed to experimental procedure. In particular Dr. Teti, Prof. Falconi and Prof. Orsini planned the research and experiments. Dr. Focaroli and Dr. Mazzotti, were responsible for cell culture, dental monomers treatment and cell viability assay. Dr. Salvatore and Dr. Teti performed western blot analysis. Prof. Ruggeri and Dr. Mattioli Belmonte performed TEM analysis; Prof. Falconi and Prof. Orsini oversaw the whole research. All authors equally and competently contributed to the draft.

## Funding

This work was supported by Fondazione del Monte di Bologna e Ravenna.

## Ethical statement

All patients provided their informed consent to participate in the study in accordance with the Declaration of Helsinki. Since the study did not expose the subjects to any risk and in agreement with the Regione Emilia Romagna and the University of Bologna Ethical Committees, instead of a written agreement form, a verbal consent was obtained from all the recruited patients. It was highlighted to all subjects that the tissue used for the study represents the usual surgical discard and that the nature of their participation in the study was entirely voluntary (freedom from coercion or undue influence, real, or imagined). Patients had sufficient opportunity to ask questions and consider their choice. The University of Bologna ethical committee approved this study.

### Conflict of interest statement

The authors declare that no one and no institution at any time receive payment or services from a third party for any aspect of the submitted work. They also declare that there are no financial relationships with entities that could be perceived to influence, or that give the appearance of potentially influencing what has been written in the submitted work. They also declare that no patents and copyrights, whether pending, issued, licensed, and/or receiving royalties relevant to the work. Finally, the authors declare that there are no relationships or activities that readers could perceive to have influenced, or that give the appearance of potentially influencing what has been written in the submitted work.
